# Analysis of mutations in DNA damage repair pathway gene in Chinese patients with hepatocellular carcinoma

**DOI:** 10.1038/s41598-022-16604-6

**Published:** 2022-07-19

**Authors:** Jiarong Li, Nianfeng Li, Muhammad Salman Azhar, Ling Liu, Liheng Wang, Qi Zhang, Langqing Sheng, Jianhua Wang, Sijia Feng, Qixuan Qiu, Yao Xiao

**Affiliations:** 1 Department of General Surgery, Xiangya Hospital, Central South University, Changsha, 410008 China; 2 National Clinical Research Center for Geriatric Disorders, Xiangya Hospital, Central South University, Changsha, 410008 China; 3 International Joint Research Center of Minimally Invasive Endoscopic Technology Equipment and Standards, Changsha, 410008 China

**Keywords:** Cancer, Genetics, Immunology, Biomarkers

## Abstract

The incidence of hepatocellular carcinoma (HCC) has increased in these years. DNA damage repair (DDR) pathway is required in response to DNA damage Gene mutations in DDR pathway play an important role in different stages of tumorigenesis and development. Based on the importance of DDR pathway in precision therapy of multiple cancers, we analyzed DDR gene mutations in Chinese patients with HCC. The results showed that (tumor mutation burden) TMB was significantly higher in HCC patients who carried somatic mutations in DDR than in non-carriers, and TMB in patients with DS, MMR mutations and DDR genes mutations such as RAD50, MLH1, MSH2, CHEK2 was significantly higher than that in wild-type patients. Based on the results of next-generation sequencing (NGS) testing, we are trying to provide clues for targeted therapy and provide feasible basis for PD-1/PD-L1 immune checkpoint inhibitor therapy.

## Introduction

Primary liver cancer is the second leading cause of tumor-related deaths and the fourth most common malignancy in China; it possesses a significant threat to the health and life of Chinese people^1^. Primary liver cancers include hepatocellular carcinoma (HCC), intrahepatic cholangiocarcinoma (ICC) along with other rare types. In most countries, HCC comprises approximately 80% proportion of total cases in histological types of liver cancer^2^. Because the early symptoms are not obvious and the disease progresses quickly, the vast majority of patients with HCC have already missed the chance of surgery when they are diagnosed^3^. In recent years, the development of targeted therapy and immune therapy improved survival of patients with advanced HCC. Even so, the highly heterogeneity of HCC makes lots of discrepancy in prognosis and treatment response between those patients with the same clinical stages. Fortunately, the progress of gene sequencing technology gives us a better understanding of the molecular characteristics of this disease, which is closer to the essence of HCC tumor biological characteristics, allows us optimize out more precise diagnosis and appropriate treatment options^4^.

Most cases of HCC were caused by viral hepatitis in China, and about 90% of them have hepatitis B virus infection. DNA damage was significantly associated with the majority of HCC cases caused by hepatitis viruses^5,6^. DDR pathway is required in response to DNA damage Genes. Different DDR pathways deal with the various types of DNA damage such as: homologous recombination repair (HRR), mismatch repair (MMR), base excision repair (BER), nucleotide excision repair (NER), nonhomologous end-joining (NHEJ), checkpoint factor (CRF), Fanconi Anemia (FA)^7,8^. DDR pathway is essential for normal cell replication and metabolism. Defects in the DDR pathway can lead to genomic instability, leading to the development of a variety of diseases, including tumors^9^. In breast cancer, prostate cancer, urothelial cancer and other cancers, it has been reported that patients with deleterious mutations in the DDR gene are more sensitive to platinum-based chemotherapy^10–12^. In addition to affecting the sensitivity of chemotherapy, DDR pathway has also become the target of anti-tumor drugs, among which the most widely studied class of targeted drugs are the poly-ADP-ribose polymerase (PARP) inhibitors, including Olaparib, Niraparib, etc.^13^. In terms of immunotherapy, it has been reported that DDR pathway are associated with high tumor mutation burden (TMB) in both ovarian cancer and non-small cell lung cancer^14,15^. Progression-free survival (PFS) and overall survival (OS) of patients with DDR pathway deficiency in urothelial carcinoma who received PD-1/PD-L1 immunocheckpoint inhibitors were longer than those of wild-type patients^16^.

## Results

### Population characteristics and incidence of DDR pathway mutations in HCC patients

In this study, 1427 patients with hepatocellular carcinoma (excluding concomitantly other cancers) who underwent surgery in our institution in the past 5 years confirmed by pathology were included, of whom 86.8% were male and 30.9% were over 60 years old. We first analyzed the mutation of DDR gene. Among these patients, 18.8% carried somatic mutations in DDR gene, and only 3.5% carried germline DDR gene mutations. Among to DDR pathway, HRR pathway had the highest mutation rate, which was 6.1%.The second was double-strand (DS), FA and MMR, with mutations of 3.2%, 2.5% and 2.2%, respectively. The incidence of mutations in NER, BER and NHEJ pathways was relatively low (0.3%, 0.6% and 0.6%, respectively) (Table 1).

**Table 1:** Population characteristics and incidence of DDR pathway mutations in hepatocellular carcinoma patients.

	Overall (N = 1427)		Overall (N = 1427)
**Age**		**MMR**	
< 60	986 (69.1%)	wt	1396 (97.8%)
> = 60	441 (30.9%)	mut	31 (2.2%)
**Sex**		**NER**	
Female	188 (13.2%)	wt	1423 (99.7%)
Male	1239 (86.8%)	mut	4 (0.3%)
**tmb_level**		**BER**	
N-Miss	92	wt	1419 (99.4%)
Mean (SD)	7.899 (5.835)	mut	8 (0.6%)
Range	0.000–83.818	**NHEJ**	
**sDDRmut**		wt	1418 (99.4%)
wt	1159 (81.2%)	mut	9 (0.6%)
mut	268 (18.8%)	**FA**	
**gDDRmut**		wt	1391 (97.5%)
wt	1377 (96.5%)	mut	36 (2.5%)
mut	50 (3.5%)	**DS**	
**HRR**		wt	1381 (96.8%)
wt	1340 (93.9%)	mut	46 (3.2%)
mut	87 (6.1%)	**OTH**	
		wt	1295 (90.7%)
		mut	132 (9.3%)

### DDR pathway gene mutations types in HCC

**Figure 1:**
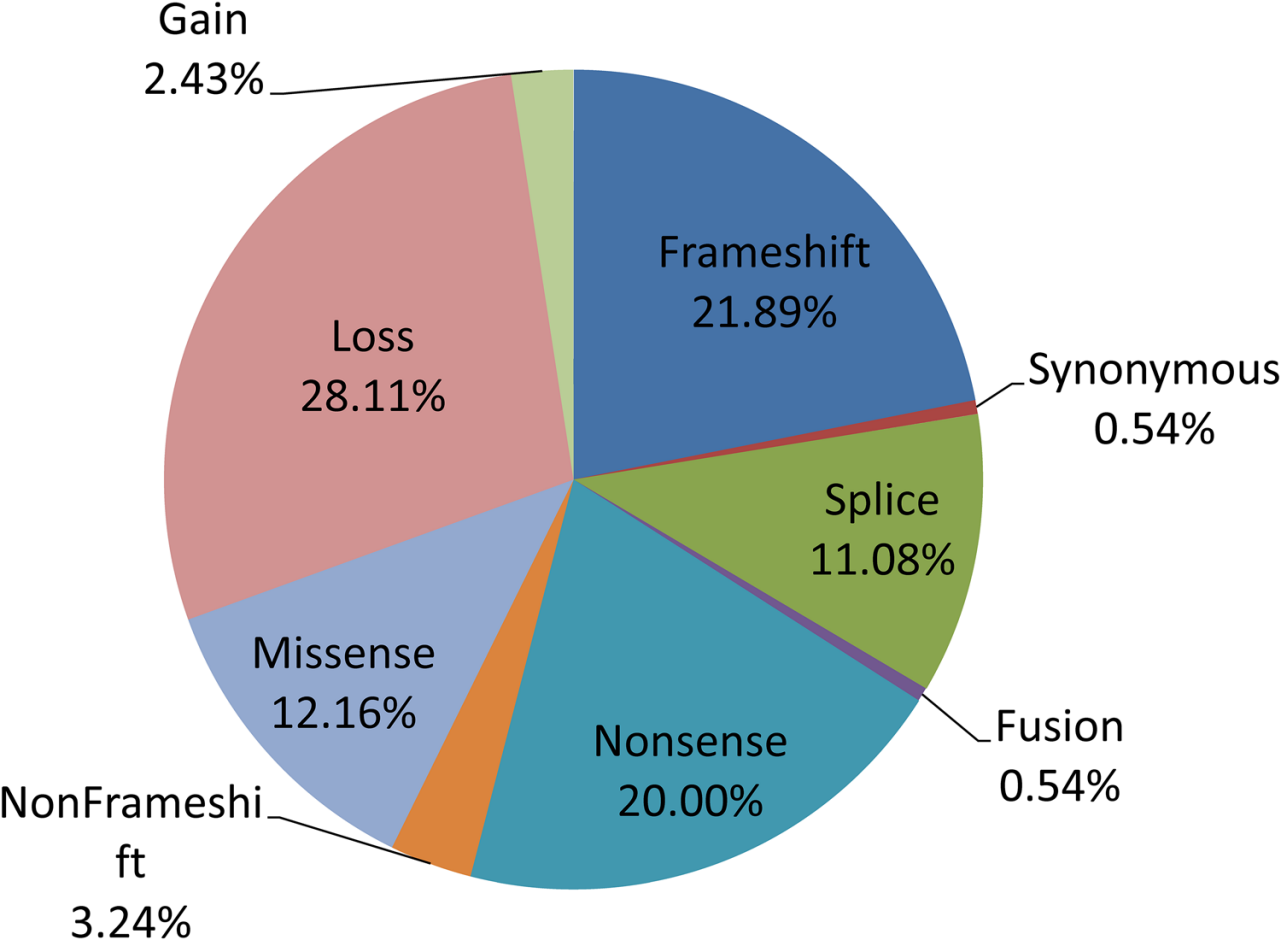
The statistical figure for DDR pathway gene mutations types in hepatocellular carcinoma.

Statistical analysis showed that the three most common types of mutations in DDR pathway genes were Loss (28.11%), Frameshift (21.89%) and Nonsense (20%). (Fig. 1).

### Frequency of DDR pathway gene mutations in HCC

**Figure 2:**
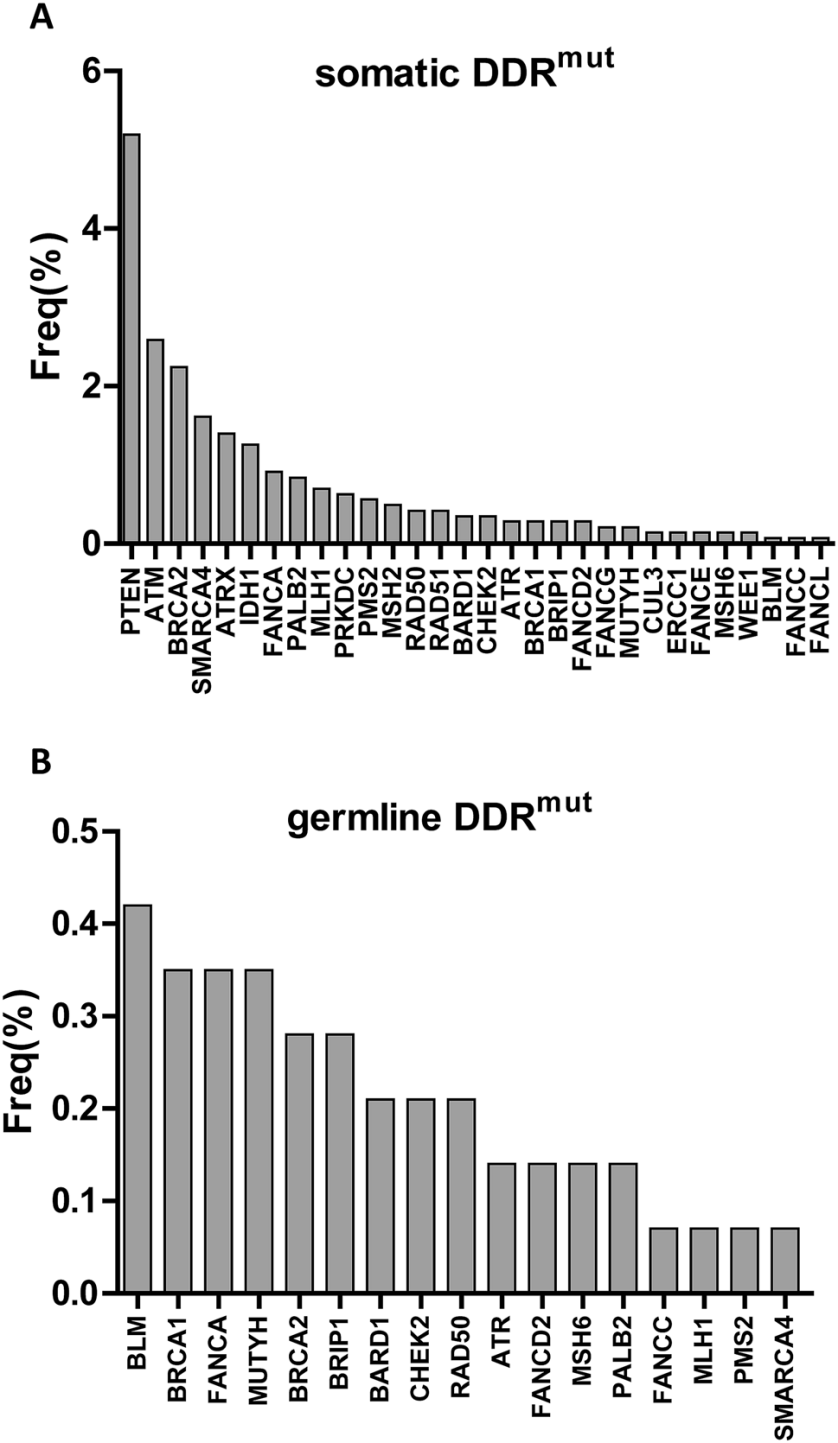
Frequency of DDR pathway gene mutations in HCC. (**A**) The gene with the highest incidence of somatic mutations. (**B**) The gene with the highest incidence of germline mutations.

In terms of mutation frequency of a single gene, the gene with the highest incidence of somatic mutations was PTEN (5.2%), followed by ATM (2.6%), BRCA2 (2.2%), SMARCA4 (1.6%), ATRX (1.4%), IDH1 (1.26%), FANCA (0.9%), PALB2 (0.8%) and MLH1 (0.7%) (Fig. 2A).Compared with somatic mutations, the incidence of germline mutations was lower, less than 1%, and the genes with higher mutation frequency were BLM (0.42%), BRCA1 (0.35%), FANCA (0.35%), MUTYH (0.35%), BRCA2 (0.28%) and BRIP1 (0.28%). (Fig. 2B).

### Correlation between DDR gene mutation and TMB in HCC patients

**Figure 3:**
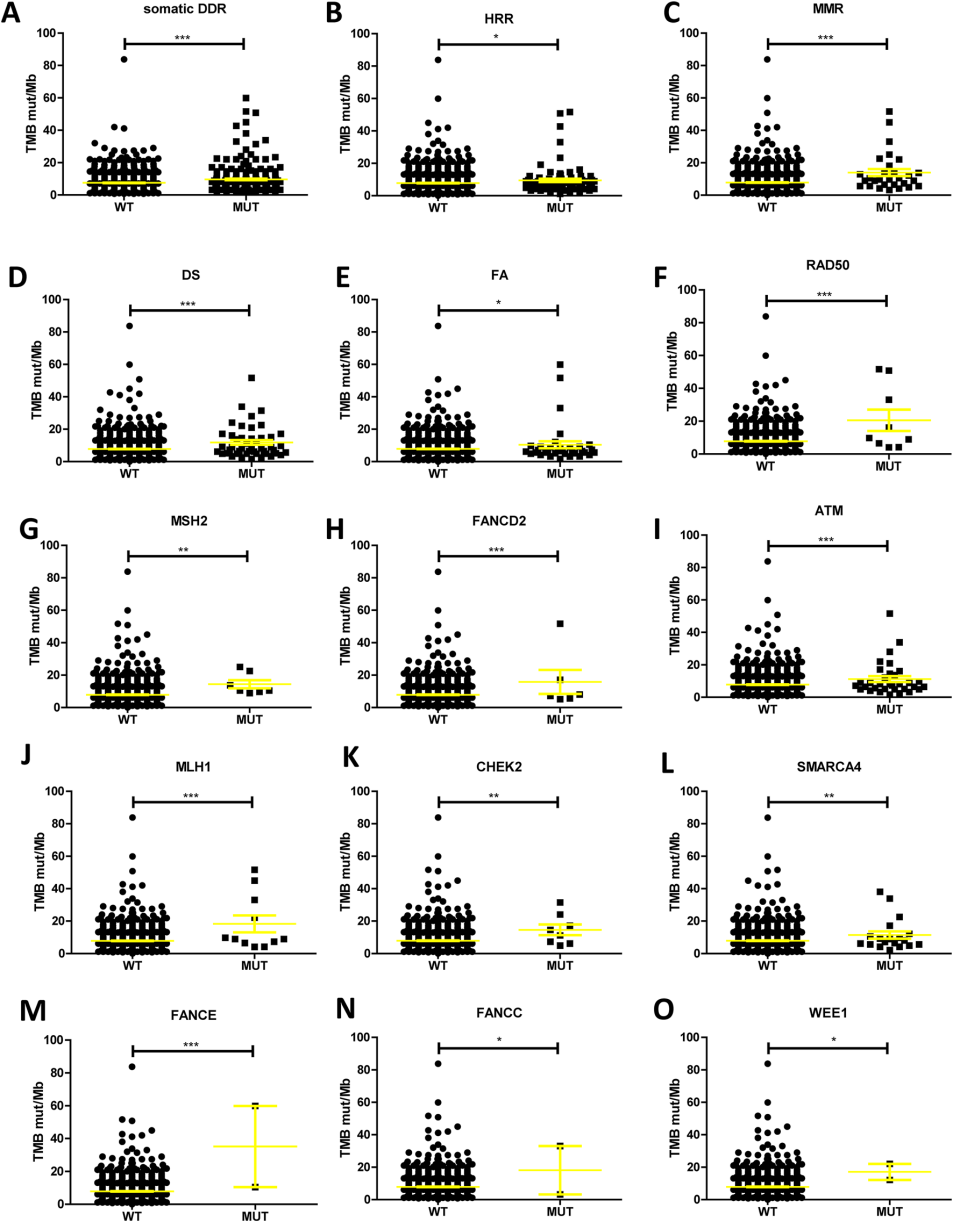
Correlation between DDR gene mutation and TMB in HCC. (**A**) The association between somatic DDR mutations and TMB. (**B–E**) By pathway analysis, the associations between HRR, MMR, DS, FA and TMB. (**F–O**) By single gene analysis, the associations between RAD50, MSH2, FANCD2, ATM, MLH1, CHEK2, SMARCA4, FANCE, FANCC, WEE1 and TMB. All quantitations are presented as mean ± SD and p values calculated by t-test, *p < 0.05, **p < 0.01, ***p < 0.001.

TMB data was available in 1335 patients with a median TMB of 7.899 (0–83.818) Mut/Mb. We analyzed the association between DDR mutation and TMB, finding that TMB was significantly higher in HCC patients who carried somatic mutations in DDR than in non-carriers (Fig. 3A), while the TMB of HCC patients with DDR germline mutations did not differ in this study (Figure S1). Analyzing the effect of DDR mutations on TMB by pathway, showed that TMB in patients with HRR, MMR, DS and FA mutations was significantly higher than that in wild-type patients (Fig. 3B–E). Patients with NER and BER pathway mutations also showed a higher trend in TMB, but the difference haven’t any statistical significance (Figure S2).

The results of single gene analysis showed that mutations of DDR genes such as RAD50, MSH2, FANCD2, ATM, MLH1, CHEK2, SMARCA4, FANCE, FANCC and WEE1 were significantly correlated with TMB elevation (Fig. 3F–O).

## Discussion

Hepatocellular carcinoma (HCC) is the second most common cause of death from cancer worldwide that often occurs in chronic liver disease and liver cirrhosis^17^. With the development of gene sequencing technology and precision medicine, more and more evidence shows the need for a genetic explanation of the development of cancer^18^. In recent years, DDR pathway has attracted more and more attention from clinical medicine and translational medicine due to its driving role in tumor genesis and guiding role in tumor precision therapy. DDR pathway has been extensively studied in breast cancer^19^, ovarian cancer^20^, prostate cancer^21^, pancreatic cancer^22^ and other cancers, while its guiding significance in the diagnosis and treatment of liver cancer remains to be further clarified.

In this study, we statistically analyzed the incidence of DDR pathway gene mutations in Chinese patients with HCC, and the results showed that about 20% of Chinese patients with HCC carried somatic or germline mutations of DDR pathway gene, among which the DDR genes with the highest mutation frequency were ATM and BRCA2, which is consistent with the previous conclusions of Lin J, et al.^23^. This suggests that the precise treatment of DDR pathway involves a considerable number of HCC patients, and the targeted therapy of PARP inhibitors and the immunotherapy of PD-1/PD-L1 have a broad prospect in the study of HCC patients.

DDR gene mutation will interfere with the cell repair DNA damage, which will lead to the accumulation of genomic mutations, leading to the increase of TMB. We analyzed the correlation between DDR pathway gene mutation and TMB in hepatocellular carcinoma. Different from previous literature^23^, somatic and germ line mutations were analyzed separately this time. Results showed that the DDR pathway in patients with somatic mutation TMB was significantly higher than that of wild type patients, while in the other existing multiple cancer types the DDR embryo mutations in patients with TMB did not show differences in the analysis, it may be that the embryo has a lower incidence of mutation, the incorporated in the analysis of DDR embryo of mutations in patients with limited number, the follow-up to further expand the sample size is needed to study. According to different points of DDR pathway analysis results showed that the DS and MMR pathway gene mutations can lead to a significant rise in TMB. Patients with mutations in HRR, BER, NER pathways also showed a higher trend in TMB, but it did not reach statistical significance in this study. Analysis of a single DDR gene showed that mutations in MLH1, MSH2, CHEK2, FANCE and other genes led to a significant increase in TMB. For other DDR channel and the correlation between gene mutation and TMB, further expansion of the sample size is needed to confirm. In addition, there is literature put forward different DDR genetics change the TMB on different weights^24^, and this view may also be a reasonable explanation for our results. Another study showed that multiple mutations in the genes of the DDR pathway caused higher TMB levels, which resulted in longer OS. By contrast, OS was significantly longer in patients with non-DNMT3A mutations than in those with DNMT3A variants. DNMT3A alteration in NSCLC patients led to poor outcomes^15^. The weight of the influence of different DDR genes on TMB is also worth further exploration in future studies.

Our study has the largest sample size of the DDR pathway in HCC patients to date which showed the gene mutation map of DDR pathway in Chinese patients with HCC. It highlighted the prospect of the clinical transformation of PARP inhibitors and PD 1/L1 immunotherapy in patients with primary liver cancer, and laid a foundation for the precise treatment of liver cancer patients.

## Materials and methods

### Clinical sample collection

Hepatocellular carcinoma (HCC) tissue specimens excised by clinical surgery and confirmed by pathology and paired peripheral blood specimens were included in this study. Tissues were made into sections, stained by H&E and independently reviewed by two pathologists to ensure that the tumor tissue volume ≥ 1mm^3^ and the tumor cell content ≥  20%.

### Next generation sequencing (NGS)

The NGS testing used to guide subsequent treatment decisions was conducted through the Clinical Gene Amplification Testing Laboratory of the College of American Pathologists (CAP) and the Clinical Laboratory Improvement Amendments (CLIA). Illumina Nextseq 500 next generation sequencing platform was used for 381 gene panel detection^25^, and the sequencing depth was at least 500× . Results included somatic and germline mutations, which were identified by comparing tumor tissue and paired peripheral blood samples.

### Statistical analysis

All statistical analyses were carried out with SPSS 19.0 (SPSS Inc, Chicago, IL). The data values were presented as the mean ± SD. Differences in mean values between two groups were analyzed by two-tailed Student’s t test. *P* value < 0.05 was considered statistically significant.

### Ethics approval and consent to participate

All experimental protocols were approved by Ethics Committee of Xiangya Hospital of Central South University. All methods were carried out in accordance with relevant guidelines and regulations. Informed consent was obtained from all participants or, if participants are under 16, from a parent and/or legal guardian.

## Supplementary Information

Supplementary Information.

## Data Availability

We understand that the data should be FAIR. However, there are several limitations to making data publicly available, as follow: This work is a retrospective study, and the informed consent did not mention making data publicly available, which would compromise patient confidentiality. The NGS test was performed in 3D Medicines Inc. Because sequencing data contains sequencing algorithm and other core trade information, it is not convenient to provide. The raw data that support the findings of this study are available from the corresponding author upon reasonable request.
