# Supplementary material for: Analysis of mutations in DNA damage repair pathway gene in Chinese patients with hepatocellular carcinoma
Source: Sci Rep. 2022 Jul 19;12:12330. doi: 10.1038/s41598-022-16604-6 (PMC9296649; doi:10.1038/s41598-022-16604-6)
Supplement: Supplementary file 1 — Supplementary Information. [file 41598_2022_16604_MOESM1_ESM.pdf]

Figure S

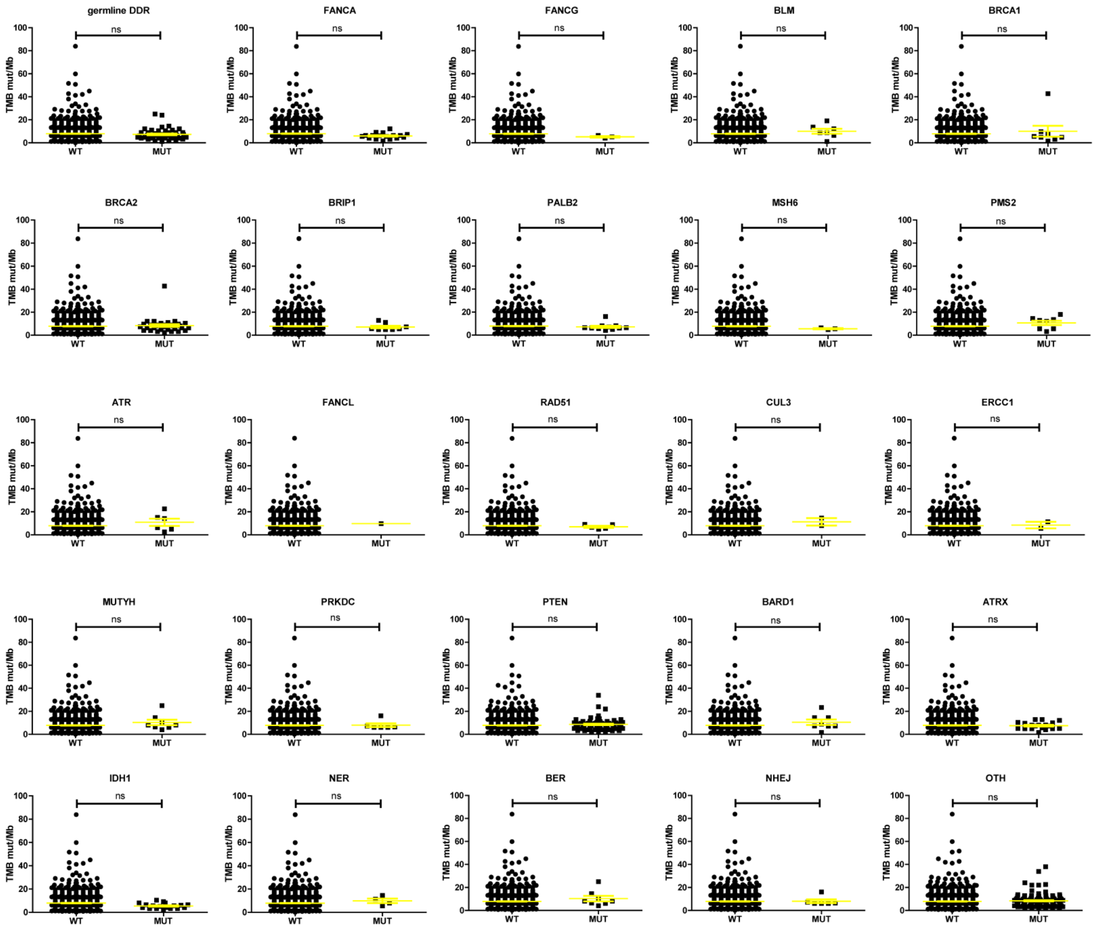

Figure S. Negative data. The associations between germline DDR, FANCA, FANCG, BLM, BRCA1 or other genes mutations and TMB. All quantitations are presented as mean ± SD and p values calculated by t-test, ns = not significant.
